# Revolutionizing Malaria Vector Control: The Importance of Accurate Species Identification through Enhanced Molecular Capacity

**DOI:** 10.3390/microorganisms12010082

**Published:** 2023-12-31

**Authors:** Mzwandile Thabani Hadebe, Samson Anjikwi Malgwi, Moses Okpeku

**Affiliations:** Discipline of Genetics, School of Life Sciences, University of KwaZulu-Natal, Westville, Durban 4000, South Africa

**Keywords:** malaria, malaria vectors, morphological classification, malaria vector control

## Abstract

Many factors, such as the resistance to pesticides and a lack of knowledge of the morphology and molecular structure of malaria vectors, have made it more challenging to eradicate malaria in numerous malaria-endemic areas of the globe. The primary goal of this review is to discuss malaria vector control methods and the significance of identifying species in vector control initiatives. This was accomplished by reviewing methods of molecular identification of malaria vectors and genetic marker classification in relation to their use for species identification. Due to its specificity and consistency, molecular identification is preferred over morphological identification of malaria vectors. Enhanced molecular capacity for species identification will improve mosquito characterization, leading to accurate control strategies/treatment targeting specific mosquito species, and thus will contribute to malaria eradication. It is crucial for disease epidemiology and surveillance to accurately identify the *Plasmodium spp.* that are causing malaria in patients. The capacity for disease surveillance will be significantly increased by the development of more accurate, precise, automated, and high-throughput diagnostic techniques. In conclusion, although morphological identification is quick and achievable at a reduced cost, molecular identification is preferred for specificity and sensitivity. To achieve the targeted malaria elimination goal, proper identification of vectors using accurate techniques for effective control measures should be prioritized.

## 1. Introduction

Malaria is a potentially lethal illness brought about by a malaria parasite, *Plasmodium spp.*, transferred to humans via the bite of *Plasmodium*-infected female *Anopheles* mosquito vectors [1]. Malaria has been previously reported as being one of the world’s most significant causes of death [1]. However, early detection and management can help minimize detrimental effects [2]. The burden is highest in sub-Saharan Africa (SSA) and several Asian nations, with rising concern of possible importation into other parts of the world, including 42 developed countries where malaria has been eradicated.

To conduct successful studies and surveillance programs for vector management, precise classification of species is vital [3]. The predominant vector in sub-Saharan Africa is the *Anopheles* mosquito, comprised specifically of *Anophele fenestus, Anophele gambie, and Anophele arabiensis*. Proper identification, classification, and accurate taxonomy are beneficial in managing and planning control strategies for malaria vector elimination. However, the different mosquito types co-exist in nature; thus, the process of elimination is not simple. Early taxonomies were based on morphological identification, but this technique is subjective, error-prone, and accompanied by multifaceted limitations that come with describing different species with look-alike appearances [4].

Recent technological improvements have substantially improved malaria monitoring capabilities, with improved sensitivity and specificity, and automated and high-throughput approaches for diagnosis. These approaches greatly enhance time-limited and efficient disease treatment in endemic locations. Despite this, the genetic plasticity of malaria vectors such as *Anopheles* mosquitoes suggests that enhanced molecular capacity for species identification is vital for improved surveillance and control [3,5]. Molecular detection and species identification of deadly *Plasmodium* transmitting vectors is vital for disease epidemiology and surveillance.

The World Health Organization (WHO) listed molecular surveillance among other indices for measuring malaria elimination [6]. This includes molecular detection/diagnosis of malaria parasites and vectors to resolve taxonomy quickly. However, the capacity for molecular identification and classification of malaria vectors is very limited, particularly in sub-Saharan Africa. Due to the insufficient capacity for genetic identification in many contexts, several mosquito species have been misidentified and treated incorrectly [7]. This has led to a rise in malaria incidence and deaths in sub-Saharan Africa (SSA) [8]. This review aims to profile the need for enhanced molecular capacity for species identification to better understand malaria vectors, especially in this era of the intensified drive toward malaria elimination.

### Vectorial Biology and Behavioral Patterns That Enhance Malaria Transmission

Mosquitoes are little, primitive insects which lay their eggs in standing water. Egg, larva, pupa, and adult are the four phases that they pass through in their life cycle. Male mosquitoes eat just plant nectar, but females take blood from their victims (hosts) to produce and nourish their eggs [9] for development and reproduction purposes. The whole process can be completed for certain species in as little as 7–10 days [9].

**Figure 1:**
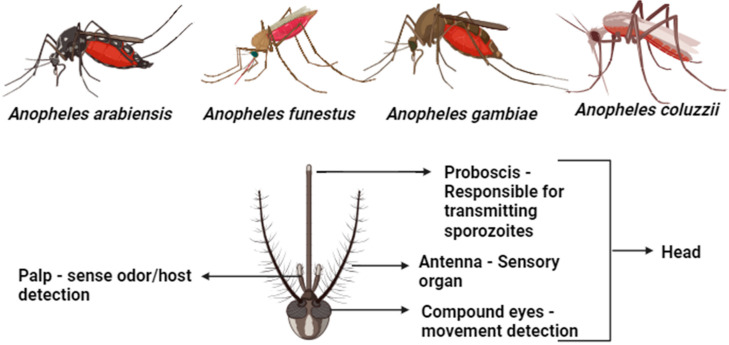
*Anopheles* spp. that are prevalent in sub-Saharan Africa, as well as a cross-section of a mosquito head displaying several organs and their roles in the transmission of malaria parasites (Created in BioRender).

Infected female *Anopheles* mosquitoes transmit *Plasmodium* species from person to person through their bites in the quest for blood meal, resulting in the insertion of sporozoites directly into the skin of the hosts, most commonly humans. Previously identified *Anopheles* complexes responsible for sporozoite insertion into human hosts in SSA include *An. arabiensis*, *An. funestus*, *An. gambiae*, and *An. Coluzzii* (Figure 1). Sporozoites are the motile infective forms of certain sporozoans produced during sporogony and commence an asexual cycle inside the new host [10]. These sporozoites go to the liver via the bloodstream, infecting hepatocytes via sinusoidal endothelial cells or Kupffer cells in the liver [11]. A parasite divides into many merozoites from the inside of a hepatocyte through a process known as schizogony. After bursting of the diseased hepatocyte, adult merozoites penetrate the circulation and infiltrate erythrocytes, initiating a new cycle of schizogony within red blood cells (RBCs), which involves asexual reproduction of their haploid genome [12]. Parasites grow in red blood cells in three stages: ring, trophozoite, and schizont. The adult schizont that results is divided and comprises 16–32 daughter merozoites. The infected RBC (iRBC) then bursts, allowing the daughter sporozoites to infect other unaffected RBCs [10]. Erythrocytic development cycles range in duration depending on the *Plasmodium spp.* present, with *P. knowlesi* taking around 24 h to mature, while *P. falciparum*, *P. vivax*, and *P. ovale* require 48 h, and *P. malariae* takes 72 h [10]. Ruptured iRBCs also produce numerous parasite metabolic byproducts, such as hemozoin, which is created when the parasite body breaks down hemoglobin.

The biology and behavioral pattern, particularly the feeding habit of mosquitoes, make them unique vectors for the *Plasmodium* parasite; as such, precise characterization of vectors causing disease is an initial step in implementing an effective control program. [13]. To focus on essential but constrained resources for managing mosquitoes, it is crucial to identify African anopheline mosquitoes based on morphology. This allows researchers to characterize parasites in the vectors and determine the chronological age of the vectors. However, morphological identification is subjective and highly prone to technical and human errors. Substantial developments in the genetic study of mosquito populations utilizing DNA-based technology are relatively new, yet effective. An amalgam of standard procedures and a battery of modern immunology and molecular testing methods can now be applied on individual specimens to produce essential biological and epidemiological knowledge [14]. Since identifying malaria vector mosquitoes accurately is crucial for eradicating and controlling the disease, enhanced molecular capacity for species identification is critical for improved surveillance and control of malaria vectors, especially in this era of intensified drive toward malaria elimination [15,16].

## 2. Distribution, Prevalence, and Control of Malaria Vectors and Challenges with Malaria Identification

Malaria generally affects tropical and sub-tropical nations, but the risk is greatest in sub-Saharan Africa, where four countries including Nigeria, the Democratic Republic of the Congo, Uganda, and Mozambique accounted for over 50% of all malaria fatalities globally in 2021 [17]. Understanding the distribution and prevalence of malaria vectors would make it easier to identify the species present in a given area and work toward eliminating them with specific treatments [14].

### 2.1. Lessons Learned from Europe

Malaria has been eradicated in Europe, as it has in the United States, but isolated imported cases [18] are still common. Malaria vectors in Europe include the *Anopheles maculipennis* complex [19]. This complex comprises ten distinct *Anopheles* species, with just three of them regarded as being the primary carriers of malaria. *An. atroparvus* is the main malaria vector in northern, western, and central Europe, whilst *An. sacharovi* and *An. labranchiae* are the main vectors in southern and southeastern Europe [19]. Other species such as *Anopheles messeae*, *Anopheles maculipennis s.s.*, and *Anopheles melanoon*, all of which are members of the *Anopheles* maculipennis complex, are minor vectors of the disease in Europe. In contrast, *Anopheles superpictus*, *Anopheles plumbeus*, *Anopheles claviger*, and *Anopheles algeriensis* play a minimal role in the transmission of *Plasmodium* in Europe [20,21]. Various *Plasmodium* parasites have been detected in Europe due to malaria being imported from various regions. Different *Plasmodium spp.* in Europe include *P. falciparum*, *P. malariae*, *P. knowlesi*, *P. vivax*, and *P. ovale* [22].

Malaria is brought into Europe by individuals who travel from malaria-endemic places or who go outside of Europe to malaria-endemic regions in search of work and then return to Europe [23]. The number of imported malaria cases increased significantly between 1972 and 1988, and malaria-positive patients increased significantly in 2000. More than 70% of these cases originated in France, the United Kingdom, Germany, and Italy [22,24,25,26,27,28]. Even though malaria was officially eliminated from Europe in 1975, the *Anopheles maculipennis* complex is still widely dispersed there [22]. Molecular identification and control of vectors in Europe is essential to malaria epidemiology and control. The need for malaria research incorporating modern methods for disease and vector control assessment, particularly in regions where malaria is widespread [14], cannot be overemphasized.

### 2.2. Lessons Learned from America

The United States of America (USA) is one of the regions that was malaria endemic. Malaria was eradicated in the USA in the 1950s [29] via enhanced treatment, sanitation, and widespread distribution of pesticides [30,31]. However, malaria vectors such as *An. Quadrimaculatus* and *An. Freeborni* still exist in the United States of America, but major vectors are not prevalent [30,32,33,34]. Imported cases triggered 63 malaria outbreaks in the United States between 1957 and 2003. Between 2011 and 2016, approximately 1773 cases of malaria were documented annually [35,36]. In 2016, there were 2078 malaria cases, which is more than the estimated number of cases identified annually from 2011 to 2016 [35]. One likely explanation for this rise in cases is malaria vector resistance to pesticides. However, according to Mace [35], most malaria cases (98.8%) were imported to the USA and about 75% were imported from sub-Saharan Africa. Airport malaria is also being blamed for the rise in the cases of malaria in the USA. Airport malaria occurs when an infectious mosquito enters a plane from a malaria-endemic region and is unintentionally transported to a malaria-free area [37,38]. Not only did imported cases increase malaria prevalence in the United States, but congenital malaria (malaria transmission from a pregnant mother to her fetus) [29] also contributed significantly to the rise in cases from 2011 to 2016.

### 2.3. Lessons Learned from Asia

Previous research has shown that these species are treated differently depending on type [39]. *Anopheles sinensis* was shown to be the most prevalent in biting people in the latest research along the north/south Korean border. At the same time, *Anopheles lesteri* was found to be the second most prevalent anopheline of mosquitoes sampled in the region [40]. These researchers found it difficult to differentiate adults of *An. sinensis* and *An. lesteri* morphologically, thus questioning the possibility of a relationship between Korean *An. lesteri* and Chinese *An. anthropophagus*, because *An. sinensis’* human biting behavior differs from that observed in China for the same species, where it mainly feeds on cattle. It was then concluded that identification by morphological features is not as accurate as molecular identification. Only *An. sinensis*, *An. lesteri*, and *An. yatsushiroensis* were identified in a recent molecular examination of samples from the Republic of Korea, despite the fact that only a limited amount of material was investigated [40].

In 1979, the Republic of Korea (ROK-South Korea) was assumed to be malaria-free [41], but in 1993, a patient tested positive for malaria caused by *P. vivax*. From 1993 until 2000, there was an annual rise in malaria cases, reaching a peak of 4142 cases in 2000. However, malaria cases due to *P. vivax* were reduced by more than half between 2001 and 2015 [42] due to strong and accurate vector surveillance, and malaria transmission by *Anopheles hyrcanus* was reduced by targeted control to destroy *Anopheles* species [43].

Malaria was prevalent in China from the 1950s to the 1970s, with around 24 million confirmed cases in 1970 [44]. Anti-malaria therapeutic control from 1980 to 2000, brought down malaria incidence by 20 cases per million persons in the year 2000 [44]. In 2010, China launched the National Malaria Elimination Programme (NMEP) to eradicate malaria by 2020 [45]. Because no indigenous cases were discovered in 2017, the NMEP succeeded in eradicating malaria in China [46]. In China, *An. sinensis*, *An. anthropophagus*, *An. minimus*, and *An. dirus* are active malaria vectors responsible for the spread of *Plasmodium*, with *An. sinensis* being widely distributed compared to other species [47,48]. The NMEP adopted the 1-3-7 approach that revolved around case reporting, investigation, and classification within 1, 3, and 7 days and increased active vector surveillance to eradicate mosquito breeding grounds and routine insecticide spray [49,50]. These notwithstanding, molecular vector species identification was very helpful in determining malaria in America and China, providing accurate identification of diverse vector species [51]. Therefore, capacity around technologies and methods used for molecular vector species identification and characterization is paramount, particularly in malaria-endemic regions.

### 2.4. Lessons Learned from Africa

In Africa, malaria is distributed by *An. arabiensis*, *An. coluzzii*, *An. gambiae*, *An. melas*, *An. Merus,* and *An. funestus*. These *Anopheles*’ species belong to different complexes with *An. arabiensis*, *An. coluzzii*, *An. melas*, *An. Merus,* and *An. gambiae* belonging to the gambiae complex and *An. funestus* specifically belonging to the funestus complex [52]. The above-mentioned Anopheline species are said to be the major vectors responsible for distribution of *P. vivax* and *P. falciparum* to humans in Africa [53]. *An. gambiae* sensu stricto is distributed widely across Madagascar and Africa [54], *An. coluzzi* is prevalent in west Africa, with transmission extending into central Africa and Angola [55], and *An. arabiensis* is widely spread throughout Africa [56], while *An. melas* and *An. merus* have been observed in west and east African coastlines, respectively [57]. *An. funestus s.s*. is thought to be the main vector regulating transmission of malaria parasites throughout southern and certain regions of east Africa; however, it is found in most African countries and can be a far more dominant vector than *An. gambiae* in other locations [58]. Additional anopheline species which transmit malaria in west and central Africa are *An. moucheti* and *An. nili s.s.* [59].

In 2015, of the 88% of the 214 million worldwide cases reported, 90% of the 438,000 deaths were recorded in Africa [60]. In 2020, sub-Saharan Africa was responsible for 95% of all malaria cases and 96% of all fatalities. Around 80% of deaths in the region occurred among children under the age of five [29]. Malaria is still highly endemic in Africa because of challenges associated with identifying vectors that belong to species complexes and the changing composition of malaria vectors. Improved molecular species identification capacity can help resolve these challenges through accurate and efficient methods of identifying vector species. This can thus encourage the advancement of strategies for the management of mosquitoes that are specifically suited to their bionomics and distribution.

## 3. Mosquito Control Strategies

Various strategies for controlling mosquito vector species have been employed globally and they continue to evolve. These include chemical insecticides that focus on the elimination of malaria vectors utilizing various kinds of chemical insecticides [61], destruction or control of the environment for larvae, larviciding with insecticides, the use of biological agents, and rotational use of insecticides to avoid the emergence of tolerance/resistance in populations of mosquitoes [62]. Innovative methods for malaria control, such as field investigations, laboratory-based research, and vector control evaluation, in endemic communities, could leverage on species identification, as well as applying the right kind of insecticide, as ways of preventing over-exposure that could result in insecticide resistance and other downstream complications.

### 3.1. Mosquito Control Strategies in Africa

Among all previously stated malaria elimination strategies, long-lasting insecticidal nets (LLINs) and indoor residual spraying (IRS) are said to be the core vector control measures in African regions, including Ethiopia. These two strategies have been proven to be effective in managing malaria but not in eradicating it. Their efficacy, however, may differ based on their specific geographical distribution, ownership, and utilization at the household level [63]. They are used to control mosquitoes that feed and rest indoors, but they cannot stop the spread of malaria outdoors, where there are effective vectors that want to eat human blood outdoors or eat indoors and relax outside [64]. Malaria vectors can develop resistance to LLINs and IRS, which is one of its limitations. Another disadvantage of the eradication procedures mentioned above is that they might result in residual transmission. Residual transmission is characterized by minimal transmission of the disease in the presence of high levels of LLINs and IRS coverage to which the local vector is completely susceptible [65]. Low transmission that remains cause malaria vectors to be resistant to LLINs and IRS. As a result, significantly increasing LLINs and IRS could result in a substantial decrease in the burden of malaria during the control phase, but it will not stop malaria transmission permanently [66].

As previously stated, apart from LLINs and IRS, there are other malaria strategies used to control malaria in Africa, e.g., improving housing, zooprophylaxis, insecticide-treated livestock, ivermectin administration to humans, odor-baited mosquito trapping systems, space spraying, ITPS, etc. Among the previously mentioned malaria vector control methods, LLINs are considered the key strategy for controlling vectors in all African regions that are endemic to malaria and are recognized to be a very successful tool in reducing malaria transmission [67]. Mosquito resistance to the pesticide chemicals on LLINs and misapplication are important issues for LLINs [68]. Recent studies found that LLINs treated with permethrin (a pyrethroid) and pyriproxyfen were more effective than LLINs fed with permethrin only [69]. According to Tiono et al. [70] and Protopopoff et al. [71], new LLINs coated with piperonyl butoxide (PBO) and pyrethroid insecticide may be helpful in combating resistance. PBO has no intrinsic pesticidal properties, but it prevents mosquitoes from producing important metabolic enzymes that mosquitoes need to detoxify insecticides before they may become poisonous. As a result, PBOs boost the effectiveness of pyrethroids on LLINs, making them more poisonous to mosquitoes.

IRS involves the application of long-lasting chemical pesticides to buildings’ interior walls and roofs to get rid of adult mosquitoes resting there [71]. Most researchers have found indoor residual spraying to help lower new malaria infections and death due to malaria. It has also been implemented as one of the most crucial techniques for controlling vectors in Africa [71,72]. According to the WHO [45], the combination of IRS and Dichlorodiphenyltrichloroethane (DDT) was essential to the achievement of the WHO-led malaria eradication campaign in the 1950s and 1960s, and it remained the cornerstone of the world’s coordinated attempt to manage and eradicate malaria today. Insecticides for IRS recommended by the WHO targeting malaria vectors include organophosphates, organochlorine (DDT), pyrethroids, and carbamates [73]. Malaria disease is increased by poor housing conditions, like exposed eaves or openings that allow mosquitoes to enter [74]. However, mosquito-proofed buildings can lower the risk of indoor malaria transmission, which happens before bedtime, by limiting mosquitoes’ entry into the residence [75]. According to Killeen et al. [76], screening and general housing improvements have been utilized in the industrialized African countries as supplements to the malaria eradication approach.

Insecticide-treated livestock refers to the treatment of animals using suitable pesticides to prevent mosquitoes from biting animals, while zooprophylaxis involves the use of animals to deflect blood-seeking mosquitoes away from the human host [76]. According to Franco et al. [77], local vector behaviors, such as zoophilic and exophilic vectors, habitat barriers among human and animal quarters, and enhancing zooprophylaxis using pesticide treatment of animals or co-intervention of LLINs and/or IRS, are all factors that influence the success of these treatments. Ivermectin (IVM) is a medication which is normally utilized to cure lymphatic filariasis and onchocerciasis. It is an effective treatment against various parasites and vectors [78].

Previous studies have demonstrated that IVM kills *Anopheles* mosquitoes that eat human blood while also helping to kill *Plasmodium* parasites as the same time [79,80]. A study conducted in the Greater Mekong sub-region has consistently demonstrated that IVM mass medication treatment can decrease malaria transmission [81,82]. Smit et al. [83] clarified that IVM is a safe and effective treatment at high dosages of 300 g/kg/day for three days to control exophagic or exophilic vectors. Ivermectin has been demonstrated to boost the impact of mass drug administration (MDA) with Artemisinin-based combination treatment (ACT) on malaria transmission with fewer MDA cycles, suggesting that additional IVM might maintain the influence on disease prevalence even if MDA coverage is lowered [78]. As a result, the WHO is exploring using mass IVM administration in people as a supplementary method to reduce mosquitoes biting in the outdoors.

Mosquito attractants in a synthetic scent blend can attract more mosquitoes than people and can be used to trap and kill mosquitoes [83]. This method may kill male and female mosquitoes, reducing the malaria vector population [84]. According to prior research, an odor-baited station may be used as a capture, and the contamination kills mosquitoes that escape the trapping net soon after [85]. LLINs can be supplemented with odor-baited traps, which can help minimize the transmission of malaria. Male mosquitoes gather in groups and compete for the attention of female mosquitoes, searching for a mate. Swarms are more common at sunset and in mapped sites [86]. The use of hand-held pesticide aerosol spray to attack these groupings was successful in a study by Zahar et al. [87,88]. Space spraying in buses, trains, and aircraft as they leave from malaria-endemic regions is recommended by the WHO to avoid malaria reintroduction into countries where it has been eradicated [88] and to reduce epidemics in urban areas or refugee camps [89]. In instances when LLINs or IRS cannot be deployed, repellent creams may give self-protection against biting mosquitoes.

The utilization of environmental alteration to limit mosquitoes’ nesting locations or the utilization of biological or chemical larvicidal treatments to eradicate the larval phase of mosquitoes are examples of larval control measures [89]. This might be useful for reducing the danger of vector bites in minimal disease transmission areas, especially in areas where the disease is being eradicated [88]. Biology may additionally serve an important role in mosquitoes vector control. Larvivorous fish, which nourish mosquito larvae, have been utilized in malaria control efforts across the globe [90]. The utilization of larvivorous fish to prevent malaria is a less expensive and environmentally friendlier alternative to insecticide-based approaches [90]. In India, using larvivorous fish in conjunction with indoor residual spraying and case treatment was demonstrated to be effective in malaria prevention [91]. Previously described malaria vector control methods, including IRS and LLINs, can aid in the reduction in disease transmission. Without more innovation, attaining and maintaining zero malaria transmission is impossible, especially in the presence of residual malaria transmission, pesticide resistance, and asymptomatic malaria.

The use of gene drives to replace populations of mosquitoes might be used to combat malaria. Gene drives are indeed being investigated as a potential novel method of managing malaria vectors, locusts, and other insects. They function by developing genetically engineered mosquitoes that breed with natural insects after being released into the environment [92,93]. The resulting offspring have alleles that lower populations of malaria vectors or reduce the likelihood of mosquitoes in transmitting the *Plasmodium* parasite [92,93]. As a result, Hoermann et al. [94] conducted research in which the malaria-transmitting mosquito *Anopheles gambiae* was genetically changed. Hoermann and colleagues utilized CRISPR-Cas9 technology to introduce an anti-malarial protein gene among activated genes after a mosquito consumes blood. This was done so that the full stretch of DNA may also function as a gene drive that can be passed down to most mosquitoes’ progeny. They initially inserted a fluorescent marker into the gene to allow them to monitor it in three different locations in the DNA, then removed the marker, resulting in a little change in genes [92,93]. Hoermann and colleagues bred the mosquitoes to check if they could breed effectively and remain healthy. Their findings suggest that this method of genetic alteration might result in effective gene drives. When unmodified mosquitoes were mixed with transformed mosquitoes, they transformed into gene drives without additional alterations.

### 3.2. Mosquito Control Strategies in Asia

Malaria control in Asia, particularly in southeastern Asia, is stated to rely heavily on vector management. In Asia, malaria vector management primarily focuses on four measures: insecticide spraying, insecticide-treated mosquito nets, larval control, and personal protection [95].

Indoor spraying utilizing DDT has been used to control malaria vectors in different Asian countries including Laos, Vietnam, and Myanmar [95]. Since most vectors rest outside, they are unlikely to be susceptible to IRS, which was historically the core of malaria elimination programs and was thought to be particularly successful against *An. minimus*, which used to eat and sleep indoors [96]. IRS employing DDT was shown to be very effective in killing *Anopheles sundaicus* in Asia, although *Anopheles sundaicus* in Vietnam showed DDT resistance [95]. Spraying not only helps to eradicate mosquitoes indoors, but it may also defend migrant workers camping in forests in this situation. Ultra-low-volume (ULV) spraying is usually used and is said to be very effective because numerous malaria vectors inhabit a small area and are frequently less resistant than indigenous populations [95].

*Anopheles dirus* is believed to be the primary carrier of malaria in southeast Asia, and because of its late feeding, it may be managed by insecticide-treated mosquito nets. Etofenprox-treated mosquito nets aided in reducing *Anopheles minimus*, hence lowering malaria in Vietnam [97]. Permethrin-treated nets were more effective than placebo nets in Malaysia, where parasite rates in individuals and sporozoite rates in *An. muculatus* were very low after employing permethrin-treated nets [97]. Permethrin also aided in the reduction in positive falciparum malaria cases among children below the age of two years in Indonesia [97]. Dolan et al. [98] reported that malaria was decreased by using family-sized nets or single nets treated with permethrin in a camp on the Thai–Myanmar border. Comparing permethrin-treated and untreated nets, the treated one was found to be more effective for children aged 4-15 years near the Thai–Myanmar border because it reduced malaria incidence despite failing to lower *P. falciparum* prevalence; however, it had no effect on *P. vixax* [99].

Larvivorous fish have been used as a tool to control *An. dirus* and *An. minimus* larvae in Thailand [100]. In Malaysia, tiny dams with siphons are utilized to flush streams regularly to reduce *Anopheles maculatus* larvae [101]. According to Moorhouse [102], oiling and drainage have previously proven to be effective in inhibiting the hatching of *An. maculatus* eggs. Previous studies reported that the Asian malaria vector species which is most likely to be susceptible to larval control is *An. sundaicus*. In Malaysia, *An. sundaicus* was effectively managed by regulating larvae with various control techniques such as oiling and constructing bunds with flapping valves to keep brackish water out [102]. Furthermore, fenthion larviciding, *B. thuringiensis* treatment, algae removal, and planting of mangroves have all been employed to reduce *An. sundaicus* in Indonesia [103]. Mosquito nets and window screens are widely used for defense against mosquitoes, while home pesticide sprays and repellents are also accessible. Lastly, mosquito coils have been also used to repel mosquitoes in Thailand [95].

### 3.3. Mosquito Control Strategies in the USA and Europe

Malaria had previously been eliminated in both the United States and Europe, but malaria cases were later imported from malaria-endemic areas. Because these two locations are impacted by cases imported from almost equivalent endemic places such as Africa, the method of controlling malaria vectors is similar. Mosquito larvae control is very effective in Europe and America [104]. Different regions, including the USA, Asia, Europe, etc., share some control measures such as insecticides, drainage ditches, and the incredible power of window screens [104,105]. Prior studies reported that biological management with larvivorous fish aids in larva removal, although it is less effective.

CDC [104] reported on an exceptional control measure, known as source reduction, which is usually used in the USA and Europe. Source reduction is the removal or complete elimination of mosquitoes’ breeding areas [104]. Mosquitoes require water for two stages of their life cycle; therefore, controlling standing water sources around the home is one of the most effective malaria vector control methods used in the United States and Europe because they will not have a place to lay eggs if their breeding site (standing water) is destroyed [106]. Larval homes can be removed through various methods, such as refilling depressions that collect water and draining marshes [104]. Chemical pesticides can eliminate mosquitoes whose habitats cannot be eliminated. Examples of insecticides which are commonly used in Europe and the USA include DDT, permethrin, organochlorines, pyrethroids, carbamates, organophosphates, organochlorine cyclodiene, and phenylpyrazoles [107]. However, the USA outlawed the use of DDT in 1972 [108]. Water oiling, in which oil is sprinkled on the surface of the water, killing pupae and larvae by suffocating them, has aided in managing malaria vectors in these two regions.

Proposed biocontrol agents, such as fungus or mermithid worms, parasitize and kill larval mosquitoes, but they are ineffective and rarely utilized. Similarly, mosquitoes’ fish have been generally ineffective [104]. In conclusion, the above-mentioned control techniques utilized in various regions limit malaria transmission in malaria-endemic regions worldwide since they kill the vectors responsible for spreading the parasite. Currently, there are several innovative genetic manipulation strategies for mosquito vector control that have recently been developed or are being developed that leverage on vector genetics in malaria control. Some of these are as follows: 1. population suppression strategies, such as the sterile insect technique (SIT), incompatible insect technique (IIT), and various transgene-based technologies including gene drives [109]; 2. population modification methods that attempt to modify vectors’ populations to include heritable elements that minimize or avert the spread of pathogens [14]; 3. paratransgenesis which involves the genetic modification of symbiotic bacteria living within the mosquito gut to interfere with pathogen transmission [110,111]; 4. the substitution of a vector population with disease-resistant mosquitoes and the release of mosquitoes containing a deadly gene to suppress the populations of interest; 5. molecular identification of mosquito species using DNA barcoding which can help improve the accuracy of mosquito identification and provide details about the makeup of a specific genus [112]; and 6. utilizing transposable element-based systems to transform significant vector mosquitoes [113]. These vector control technologies have been utilized to a limited extent in the distribution of disease pathogens transported by *Aedes* mosquitoes at different trial locations but are challenged by diverse ethical issues that must be overcome before general use and acceptability.

## 4. Identification and Characterization of Malaria Vectors

### 4.1. Morphological Identification of Malaria Vectors

Malaria vectors are arthropods belonging to the class Insecta and their order is Diptera. Different methods and tools are utilized for the morphological classification of malaria vectors (*Anopheles*) previously discussed [114]. Just by studying the dispersion and structure of scales on the thorax and abdomen, Theobald (1899) discovered four sub-genera of *Anopheles*: *Cellia*, *Kerteszia*, *Nyssorhynchus*, and *Stethomyia* [114]. However, Christophers et al. [115,116,117,118,119] were not satisfied with Herbach’s method of classification since, after utilizing their classification system, which focused mainly on the quantity and placements of specific setae on the gonocoxites of the male genitalia, they were able to discover new *Anopheles* sub-genera such as *Anopheles*, *Myzomyia (Cellia)*, *Nyssorhynchus*, *Stethomyia*, *Kerteszia*, *Lophopodomyia*, *Christya,* and *Baimaia* [114]. Despite the importance of morphological identification of malaria vectors, it has significant limitations/challenges, as discussed in one of the sections below.

### 4.2. Classification of Mosquitoes

Mosquito vectors are classified into three genera: *Anopheles*, *Aedes*, and *Culex* [120]. The classification of Theobald (1899) suggested distinct *Anopheles* genera based on the distribution and structure of scales on the thorax and abdomen [114]. At the time, *Cellia*, *Kerteszia*, *Nyssorhynchus*, and *Stethomyia* were accepted as sub-genera of *Anopheles*. Since new *Anopheles*’ species were discovered after Theobald’s *Anopheles*’ classification, his categorization was neither practical nor natural, and as a result, several scientists [115,116,117,118,119,120,121] questioned his method of categorization. A new classification system that emerged following this controversy focused on the quantity and placements of specific setae on the gonocoxites of the male genitalia. This categorization technique aided in the discovery of various *Anopheles* sub-genera, including *Myzomyia* (*Cellia*), *Nyssorhynchus*, *Stethomyia*, *Kerteszia*, *Lophopodomyia*, *Christya,* and *Baimaia* [114]. Until now, previously described sub-genera have been used for *Anopheles* categorization, and they contain the species that spread human malaria parasites. Most of the malaria-transmitting anophelines are members of species complexes that commonly comprise vector and non-vector species. It is difficult, if not impossible, to separate these co-existing complexes morphologically. Despite the existence of the species complexes, which make vector identification of malaria vectors more difficult, solitary *Anopheles* species frequently demonstrate high variation throughout a wide geographic range [114], making morphological identification alone a more difficult-to-use technique for differentiating malaria vectors from other mosquito species. However, a combination with molecular identification has been more successful [122] than the morphological approach alone.

### 4.3. Molecular Identification of Mosquito Species

The molecular species discrimination tool relies on the variation in recombinant DNA (rDNA) sequences [123]. Therefore, the rDNA group has become a well-known tool in atomic entomology [124] and is also used to develop diagnostic tests to distinguish cryptic Anopheline species [11]. Mitochondrial DNA (mtDNA) also varies among different species [9]. Hence, mtDNA barcode is another tool used by geneticists to distinguish mosquitoes, with the help of other genetic markers [9].

Genetic markers are the major tools used by geneticists for the identification of malaria vectors. The variety and quantity of molecular markers accessible for the research of disease vectors has nearly tripled in the previous decade [10,125]. According to Favia et al. [12], genetic markers have evolved from the “traditional tools” of polytene chromosomal cytology, genetic compatibility, immunological and hybridization procedures, and isozyme analysis to include a diverse range of molecular markers. These modern markers vary from so-called “traditional genetic markers” (mitochondrial DNA and complementary DNA (cDNA)) to techniques for detecting and identifying single nucleotide polymorphisms (SNPs) and to highly polymorphic markers (random amplified polymorphic DNA (RAPDs), microsatellite DNAs, and amplified fragment-length polymorphisms (AFLPs)) [65]. DNA taken from several malaria vectors may be analyzed using different techniques such as PCR, real-time PCR, next-generation sequencing, etc., in which an individual marker is used to analyze DNA from distinct malaria vectors or numerous markers are used to analyze DNA from a single malaria vector. However, there is still a dearth of capacity regarding trained personnel with the technical know-how for conducting molecular assays, particularly in regions where malaria is most endemic. Equipment and other infrastructures for molecular sequencing and assays are equally scarce. Where they are found on the continent, they are located so far apart that collaboration and joint research for understanding the regional status of malaria is rendered very difficult [126]. Knowing how genetically varied wild mosquito species are, how pesticide resistance develops and spreads, and the frequency and selective advantages is critical for maintaining present malaria control success and driving toward malaria eradication.

Apart from research into malaria vector classification, molecular studies of genes undergoing selection [127] and of demonstrated alterations occurring in some of the genes of *Anopheles* species conferring insecticide resistance on these vectors further shed light on our understanding of the genetic potentials of the malaria vector, which morphological classification alone will not be able to pinpoint.

### 4.4. Taxonomic Characterization of Malaria Vector Species

The goal of categorization is to group biological entities with certain common properties. Mayr and Bock [128] defined classification as “The organization of related things (objects) in a hierarchical succession of nested classes, in which each more inclusive higher-level class is divided completely into less inclusive classes at the next lower level”, and these groups (classes) are referred to as a taxon (taxa: plural form). A taxonomic rank or category is the level of a taxon in a hierarchical classification [115].

In addition to the previously mentioned classification system, various scientists, including Edwards et al. [116,123,129,130,131,132,133], developed the systems for internal categorization of the genus *Anopheles*. Their approach creates a hierarchy of informal taxonomic divisions for the three main sub-genera *Anopheles*, *Cellia*, and *Nyssorhynchus*. Depending on the structure of the pupal trumpet, the sub-genus *Anopheles* is separated into two parts. These were created for different purposes, with the Laticorn Section designed for species with a broad funnel-shaped trumpet with the longest axis transverse to the stem, while the Angusticorn Section was formed for species with a semi-tubular trumpet with the longest axis vertical and more or less parallel to the stem [130]. The sub-genus *Nyssorhynchus* is classified into three parts based on the different combinations of larval, pupal, and adult characteristics [134]. The majority of categories at each level of categorization are assumed to reflect natural species groups, indicating phylogenetic links; however, a much more fundamental taxonomic study is required before the informal and formal taxa can be established as monophyletic units.

According to Harbach [114], taxa classes must have the same phylogenetic rank, in practice; however, they are essentially subjective groupings of subordinate taxa that are assumed to represent monophyletic groups of species and are allocated to taxonomic ranks based on common physical and biological traits rather than phylogenetic equivalency. As a result, the taxonomic classifications of the genera *Anopheles*, including the formal rank of the sub-genus, must not be regarded as phylogenetic equivalents [114]. Under the International Code of Zoological Nomenclature, infra-sub-generic categories known as taxonomic ranks below the sub-genus have no official validity. They are only convenience groups, frequently based on surface resemblances that may or may not reflect natural connections. Sections, series, groups, sub-groups, and complexes are some informal groupings used in *Anopheles* categorization or classification [114].

### 4.5. Challenges Associated with Structural Species Identification

According to Erlank et al. [13], there are disadvantages to structural species identification, just as there are to other mosquito identification techniques, such as when mosquito samples have lost significant external characteristics of their anatomy (e.g., feet), which is common if using a collection process like the Centers for Disease Control and Prevention miniature light traps (CDC-LT), where mosquitoes are destroyed as they are sucked through the fan blades. Drawbacks of the morphological identification of species may also be caused by the level of the abilities required to carry out the identifications which may be insufficient or may not exist at all. Researchers in eastern Zambia recently utilized two genetic tests, COI mtDNA and ITS2 rDNA, to validate the morphology of malaria vectors [135]. They morphologically identified 8 *Anopheles* species; however, 18 species or groups were identified molecularly, 16 of which had individuals that were morphologically characterized as belonging to the *An. funestus* group and 12 of which belonged to the *An. gambiae* complex. On the other hand, certain species that were morphologically classified as both *Anopheles Funestus* and *Anopheles Gambia* Group were molecularly identified as “*Anopheles coustani*” [135]. This suggests that when identifying malaria vectors, morphological identification alone is insufficient; it should always be verified using molecular approaches that are highly specific.

Based on multiple genetic approaches, most prior research has established that practically every morphological taxon investigated thus far is a species complex, with the *An. gambiae* complex and the *An. funestus* group being the most well known [136]. Coetzee [137], however, clarified that there are other species complexes, such as *Anopheles coustani*/*crypticus,* which shows variability in chromosome, and *Anopheles nili*/*marshallii*/*letabensis*/*hughi,* which also shows variation in chromosomes [138]. *Anopheles pharoensis*, *Anopheles longipalpis*, and *Anopheles squamosus* were only molecularly categorized and are among the complexes that are yet to be morphologically characterized [135]. New mosquitoes’ species are being discovered from molecular information. However, these molecular forms should be linked to iso-female lines better to understand their role in the spread of malaria. These lines can provide details on the genetic variation within families and relevant morphological descriptions. Molecular techniques’ emergence and subsequent application to research or surveillance is based on morphological classification using dichotomous keys [11,15,139]. Because of the above-mentioned facts, it is critical to classify species using both morphological and molecular classification, beginning with morphological identification and then moving on to molecular classification, because the information mentioned above revealed that some species might be incorrectly classified morphologically due to a variety of potential limitations, such as body parts lost during collection. Due to their great sensitivity, molecular methods and markers can detect all variations in the relevant genes, hence they rarely misidentify species. Consequently, it is crucial to confirm morphological categorization with genetic classification regularly.

### 4.6. Molecular Characterization of Malaria Vectors

It is always hard to distinguish related malaria vector species morphologically because of limitations, such as losing some body parts during mosquito collection. However, it is always possible to differentiate them molecularly. As mentioned earlier, the ribosomal DNA internal transcription spacer region 2 (rDNA ITS2) and the mitochondrial DNA cytochrome oxidase sub-unit 1 (mtDNA CO1) are two rapidly evolving loci, distinguishing species complex members. They also provide the reference sequence to generate *Anopheles’* unique barcode that identifies and distinguishes species [11,140]. Despite the fact that databases are becoming more abundant with *Anopheles* sequences at these two loci, numerous common anophelines are still to be studied molecularly. According to Harbach [114], regardless of the fact that the genus *Anopheles* has almost 500 species, there are only around 200 ITS2 and CO1 sequences in GenBank (National Center for Biotechnology Information [NCBI]). Genetic testing of local anophelines, including suspected non-vector species, would allow for the correct matching of bionomic features with species, enabling adequate assessment of the effectiveness or limits of treatments being applied.

According to Kengne et al. [124], every repeating unit of rDNA in eukaryotic organisms has an intergenic spacer (IGS), followed by genes coding for the 18S, 5.8S, and 28S rDNA. The external transcribed spacer (ETS) precedes the 18S gene, while the internal transcribed spacers 1 and 2 surround the 5.8S rDNA (ITS1 and ITS2) [11]. This multigene family evolves cohesively within species through coordinated evolution, which tends to homogenize sequences within species while promoting species divergence [141]. Although non-coding DNA sequences are known to drift away quickly even among closely related species, coding DNA sections are considered exceptionally conserved even among distantly related species. As a result, by using primers located in conserved rDNA regions, variable portions from a wide variety of species can be amplified, despite the lack of prior sequence information [11]. Sequence diversity in the ITS2 region has already been observed across different Anopheline species from various countries [142,143]. Thus, ITS2, together with other markers such as COI, 5.8S, 28S, 16S-rDNA, etc, has been mostly utilized to differentiate distinct *Anopheles*’ species in various countries/regions worldwide (Table 1).

A study by Gao et al. [152] demonstrated that two different malaria vectors (*Anopheles anthropophagus* and *Anopheles sinensis*) are closely related species, which made it difficult to separate them morphologically; however, they were found to be genetically dissimilar. Their genetic dissimilarity was identified based on PCR-RFLP analysis of the *ITS2*, digested with either HinfI or RsaI. Previous research has shown that it is feasible to morphologically classify a mosquito species as another species and then receive something different while performing DNA analysis. Malaria eradication is threatened by the prevalence and widespread distribution of the malaria vector; however, since genetic markers have aided in the identification and elimination of vectors, they may be a successful way of reducing population size and limiting distribution, which may also limit disease transmission. Utilizing adult and egg morphology, malaria vector species collected in Guangdong were morphologically recognized as *Anopheles anthropophagus*; however, a few of these species were molecularly categorized as *Anopheles sinensis* [152]. After that, sequencing analysis validated the observation [152]. Next-generation sequencing [153], genotyping-by-sequencing, restriction site-associated DNA sequencing, and RNA sequencing are common sequencing techniques used in population genetics studies [154] and their strength and limitations are discussed in some sections below. Furthermore, the Liaoning material revealed considerable variation in the egg deck width. This feature has been characterized as wide, moderate, narrow, or extremely narrow and has been used to distinguish *Hyrcanus* species [131,155].

The *Hyrcanus* family includes a vast number of closely related species that may be found across the southern Palaearctic and Oriental areas, from Spain to China, Mongolia, and Russia, and along the Indonesian archipelago to East Timor, and this group is comprised of 28 species [131]. According to a study conducted by Gao et al. [152] concentrating on the deck width morphological trait, some malaria vectors from Liaoning seemed similar to *Anopheles anthropophagus* by having a narrow deck, whereas others appeared to be *Anopheles sinensis* (with a wide deck). Despite the availability of these data, the PCR-RFLP approach successfully identified all specimens obtained in Liaoning as *An. anthropophagus*. These data show that egg deck width may not be as accurate as previously assumed in distinguishing *An. anthropophagus* from *An. sinensis*.

There is a lot of merit associated with the molecular characterization of malaria vectors. Some molecular characterization techniques use allozymes or DNA, and these methods have the benefit of being usable for both genders and all developmental phases [112]. DNA-based methods of classification have substituted allozyme approaches because they have the benefit of needing fewer steps for material preservation [112]. Another advantage of the molecular characterization of malaria vectors is the potential for adapting DNA-based approaches for very inexpensive identification, much like for the *Anopheles gambiae* complex [156,157,158].

The molecular approach, specifically PCR, is so sensitive that it can identify a mosquito by using a small body component such as its leg, which is advantageous since the remainder of the body may be utilized for further analyses such as parasite identification. On the other hand, a method known as Random Amplified Polymorphic DNA (RAPD), a PCR-based method for detecting variation, exists. The big benefit of RAPD is that it may be used in systems without any prior molecular knowledge of the genome [159]. RAPD markers are frequently found in the DNA’s repetitive, highly changeable sections. If it leads to intraspecific variation, this can work against species identification but may help separate very closely related species [156]. Ribosomal DNA (rDNA) has been promoted by Collins and Paskewitz [11] for use in identifying cryptic *Anopheles* species. Ribosomal DNA is said to be a well-characterized marker. The advantage of employing rDNA for identifying mosquitoes is that the nature and scope of the changes on which an analysis is based are much more evident [112].

Despite its benefits, molecular characterization has limitations and difficulties. One of the limitations of molecular characterization is the cross-hybridization of DNA, or variations in copy number across a species’ geographical range [156,157,158]. The fact that RAPD alleles are frequently dominant [112], as well as the challenge in attributing similarity to amplified sections, might further make RAPD data challenging to interpret. The completion of some reagents during analysis causes the procedure to be delayed, forcing the investigator to take longer than intended to complete the study, and also, the reagents being expensive adds to the disadvantages of molecular characterization of malaria vectors. An additional drawback of the molecular identification of malaria vectors is that PCR-based molecular methods are time-consuming, and tiny errors during analysis might cause one to fail to obtain the essential findings, thereby delaying the characterization process.

### 4.7. Sequencing Techniques Normally Used in Population Genetics Studies, Their Strengths, and Limitations

#### 4.7.1. Next-Generation Sequencing (NGS)

**Figure 2:**
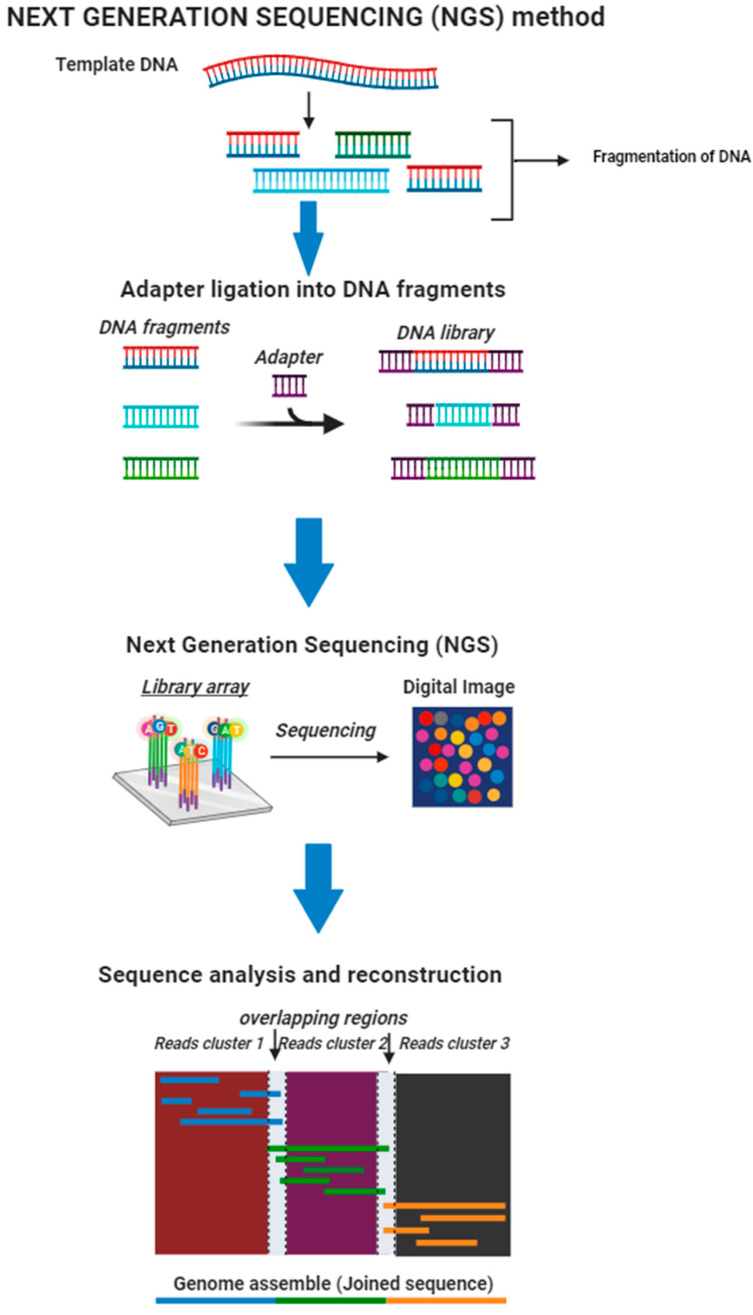
Steps involved in the next-generation sequencing (NGS) procedure (Created in BioRender).

NGS methods have changed population genetics studies by allowing for significant information gathering from chromosomes or portions of genes from a large group of individuals [153] (Figure 2). The development of NGS has significantly contributed to population genetics research by lowering prices and creating vast amounts of sequencing data [11]. The main advantage of NGS is that it may identify anomalies across the entire genome (whole-genome sequencing only), i.e., it can detect insertions, substitutions, duplications, deletions, copy number alterations (gene and exon), and chromosomal translocations/inversions [160,161]. NGS also has the advantage of identifying all of these anomalies utilizing much less DNA than older DNA sequencing methods [162]. Next-generation sequencing is also cheaper and quicker [163]. Higher sensitivities to identifying low-frequency variations [164], extensive genome coverage, and the capability of sequencing hundreds or even thousands of genes or genomic portions simultaneously are all advantages of NGS [165].

Despite NGS’s advantages, it has several limitations. The requirement for PCR amplification before sequencing is a fundamental limitation of all 2G NGS approaches [166]. Gkazi et al. [166] also clarified that PCR amplification bias during library processing and analysis is linked to this. Another limitation of NGS is the poor interpretation of homopolymers and the inclusion of erroneous dNTPs by polymerases, leading to sequencing mistakes [166]. Next-generation sequencing also necessitates advanced bioinformatics tools, rapid data analysis, and massive data storage capacities, all of which can be expensive [167]. Several universities might just have the financial resources to buy next-generation sequencing equipment but often lack the computing resources and effort to analyze and scientifically interpret the results [168].

#### 4.7.2. Genotyping-by-Sequencing (GBS)

GBS is a technique used by scientists to genotype samples and find genetic variants rapidly. This method is also called high-throughput sequencing, which uses restriction enzymes to minimize genomic complexities [169]. Although this approach is cost-effective, it generates a lot of incomplete information and necessitates a lot of genetic analysis [170]. According to Mukherjee [171], genotyping-by-sequencing’s key strengths are its sensitivity, speed, and ability to identify minimal background signals. Possible disadvantages of GBS include a high proportion of missing data points due to limited sequencing depth and the handling and interpretation of enormous amounts of sequence information [172].

#### 4.7.3. Restriction Site-Associated DNA Sequencing (RAD-Seq)

**Figure 3:**
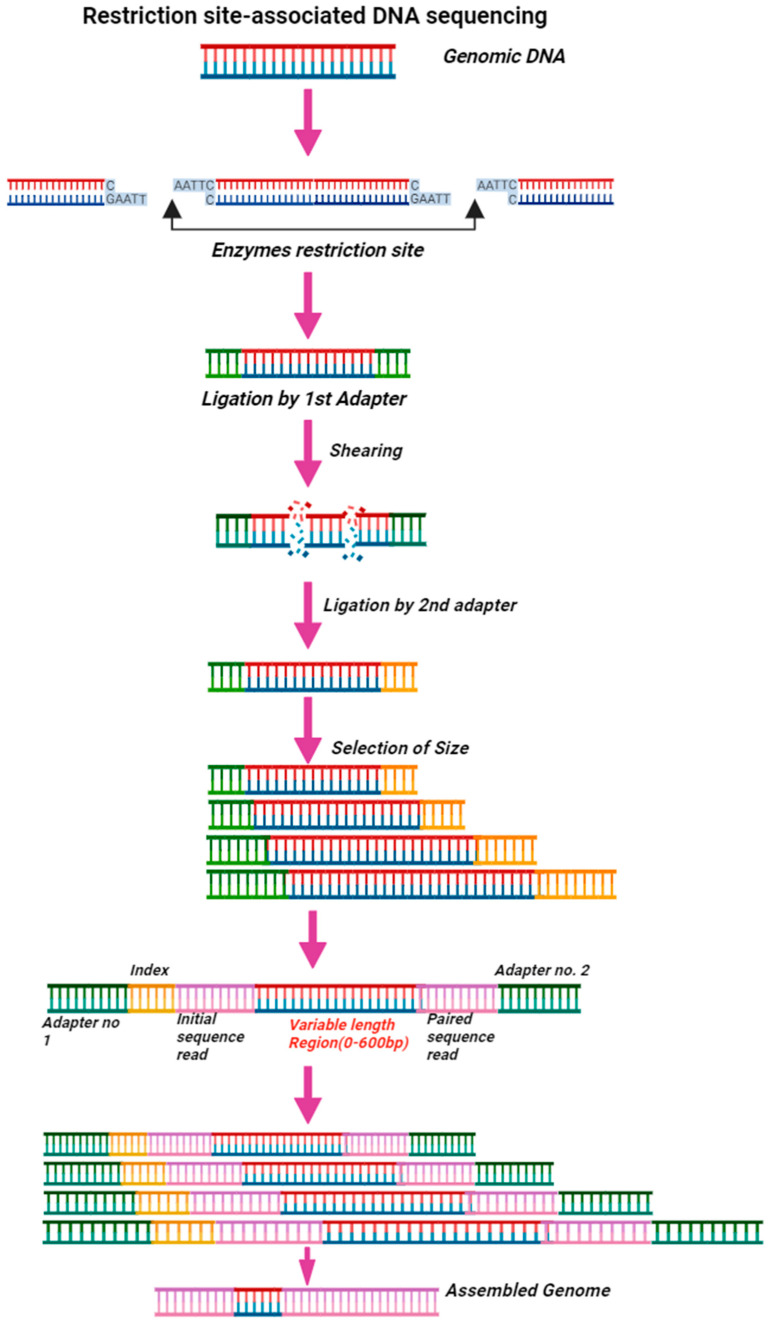
Step by step process of Restriction site-associated DNA sequencing (RAD-Seq) (Created in BioRender).

The RAD-Seq technique is based on the restriction of DNA sequences utilizing a single restriction enzyme (Figure 3). This approach is very flexible when selecting a restriction enzyme to attain the desired reduction factor. As a result, RAD-Seq may sequence a small number of loci or many sites at lower or higher coverage [173]. Previous studies have clarified that RAD-Seq’s principle has been utilized in GBS [174] and double digest RADseq [175]. Because of their flexibility and cost-effectiveness, both GBS and RADseq methods have been widely employed in population genetics in various species, including mosquitoes [176]. RADseq is comparable to RFLP (fragment-length polymorphism) and AFLP (amplified fragment-length polymorphism) examinations because it also minimizes the intricacy of the genomes by subsampling only at particular regions designated by restriction endonucleases [177]. Compared to these techniques (RFLP and AFLP), RADSeq has the advantage of simultaneously discovering, confirming, and evaluating markers and reliably identifying which markers originate from each location [177]. RADseq may be employed in wildlife populations and on crosses of any design, allowing for not just sequencing and SNP detection but also more advanced analysis, including quantitative genetics investigations [177]. RADseq is frequently utilized because of the strengths mentioned above; however, it does have certain limitations or drawbacks. The fundamental weakness of this technique is that there is very little control as to which sections of the genome are analyzed, and sequence density is randomly distributed over the genome, making either one area of interest poorly covered [178].

#### 4.7.4. RNA Sequencing (RNA-Seq)

**Figure 4:**
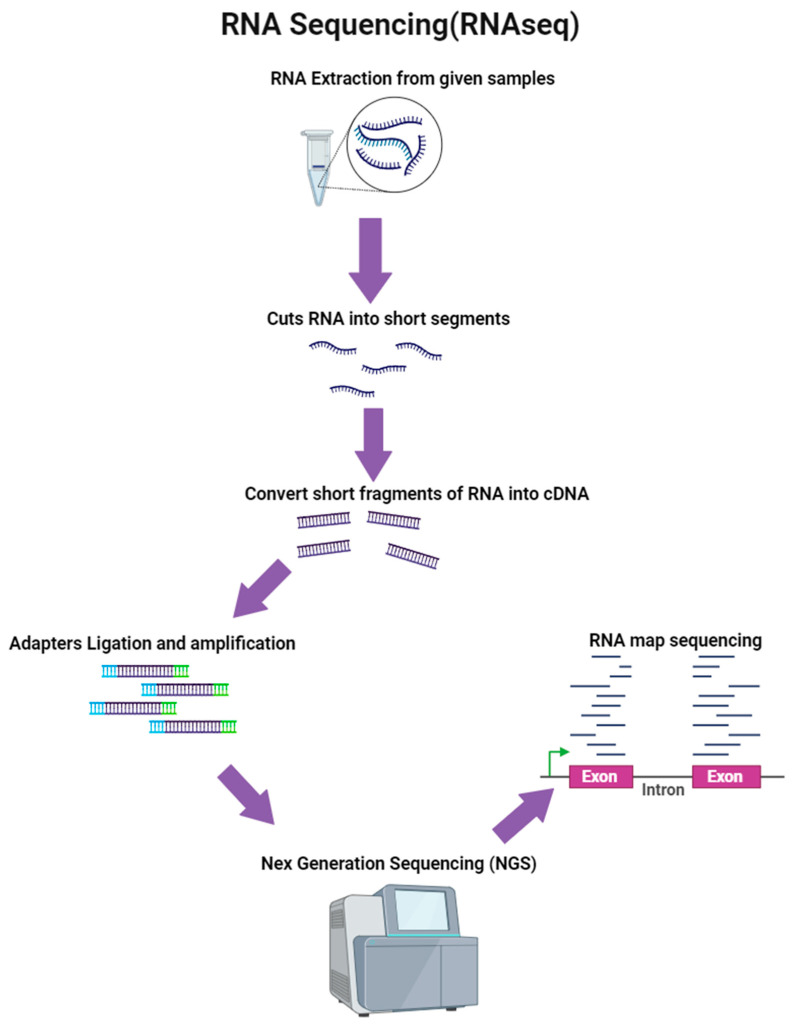
Step by step process of RNA sequencing (RNA-Seq) (Created in BioRender).

RNA-seq is a method that uses NGS to examine the quantity and sequences of RNA in a sample [179] (Figure 4). This method aids in investigating and discovering both known and novel features in a single assay. According to Ozsolak et al. [180], the strength of RNA-seq is that it can determine which genes are active inside a cell, their transcription levels, and when they are active or inactive. Other advantages of RNA-seq are that it allows for direct sequence alignment (no hybridization) and the identification of paralogs, and it can be used to identify SNPs. It does not rely on existing sequence data, and alternative splicing is found if somehow the sequence is matched to the genome [181]. Regardless of the strengths RNA-Seq mentioned above, it also has a number of limitations or drawbacks. According to Martin et al. [181], RNA-seq’s limitations include high costs, the need for high-power computer facilities, analysis that can be tricky if paralogues are present, making the study of splice variants hard, and a high set-up cost if conducted in-house.

In addition to the advantages listed above for each sequencing approach, the major advantage of sequencing techniques in general is their ability to identify any alteration in the genome or in the genes of interest, thus enabling the study of genetic diversity observed in the many genes of various mosquito species under investigation. Due to their great sensitivity, they are the primary tool used by scientists to examine the genetic variation in mosquitoes and to validate the morphological characterization of mosquito vectors. The cost of the polymerase chemicals required for the DNA polymerase enzymatic process and the high error rate with duplicated nucleotide insertion during variant identification are the main challenges for the aforementioned sequencing procedures [182]. An additional difficulty with sequencing methods is that they might generate huge datasets, which poses a significant algorithmic barrier when attempting to match the data to a reference genome [183].

Together, these benefits significantly contribute to eradicating malaria because they allow for the production of molecular characterization data, which improves our understanding of the genetic variety of malaria vectors and the genes that induce pesticide resistance in mosquitoes. Thus, chemists or other medical professionals can use the information gathered to develop insecticides that can kill mosquitoes that contain pesticide-resistant genes.

### 4.8. Capacity for Molecular Identification and Characterization of Malaria Vectors in Sub-Saharan Africa

**Figure 5:**
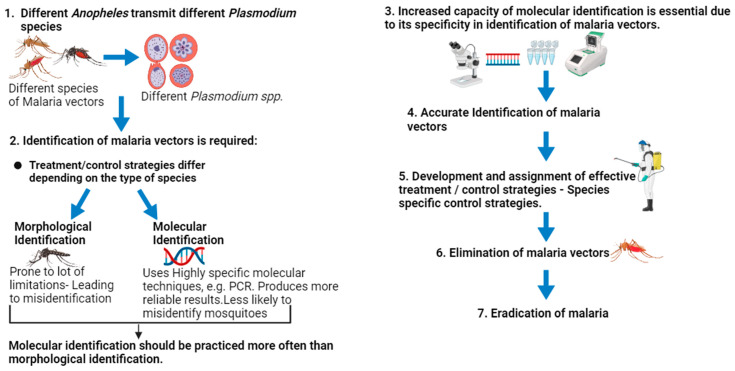
Emphasizing the need for increased capacity for molecular identification of malaria vectors (Created in BioRender).

Molecular characterization of malaria vectors involves the utilization of highly specific equipment and procedures such as DNA extraction kits, PCR, nucleotide sequencing, nucleotide comparison on GenBank, multiple sequence alignment (MSA), and phylogenetic analysis [184]. In addition to the information above, genetic markers—highly specialized tools—are essential for the molecular characterization of mosquitoes, since earlier studies showed that these gene regions vary depending on species. By focusing on these specific regions, researchers successfully identified distinct species of malaria vectors, analyzed their genetic linkage, and uncovered novel haplotypes [143,185]. Cytochrome oxidase c sub-unit I (COI), cytochrome oxidase c sub-unit II (COII), internal transcriber spacer 2 (ITS2), 18S rDNA, and 28S ribosomal DNA are the most frequently used genetic markers for characterization of malaria vectors [147,186]. It is crucial to note that, despite being extremely precise, molecular characterization techniques are time-consuming and expensive, which is the reason why they are not used more frequently in areas of Africa where malaria is prevalent. As a result, most previous research seeking to prevent malaria by characterizing mosquitoes relied more on morphological characterization than molecular characterization [187]. A prior study, however, showed that morphological characterization is susceptible to several restrictions that make it difficult to correctly identify malaria vectors, which results in the incorrect treatment of malaria vectors in specific regions [188]. Some African regions where malaria is endemic include Nigeria, the Democratic Republic of the Congo, Uganda, Mozambique, Zimbabwe, and South Africa [189]. There have been relatively few studies based on the molecular characterization of mosquitoes in the locations mentioned above. The majority of studies focused on the characterization of mosquitoes are based on their morphology. Therefore, the limited capacity for the molecular characterization of malaria vectors in RSA and other malaria-endemic regions of Africa has contributed to a rise in malaria incidences and fatalities associated with malaria as a result of the rise in the spread of mosquitoes; hence, this calls for a need to increase the capacity for molecular identification and characterization of malaria vectors (Figure 5). This basically suggests that in order to minimize the transmission of malaria vectors, scientists must perform more molecular characterization of vectors or always confirm morphologically characterized species with molecular techniques to guarantee that suitable treatment is developed and administered to specific vectors prevalent in a particular location. This will undoubtedly decrease the spread of malaria vectors, hence decreasing the transmission of malaria parasites.

There are several ongoing studies on the molecular identification and categorization of malaria carriers in sub-Saharan Africa (SSA), as evident in the published literature. Some of the studies include molecular technologies and genetic methodologies developed to investigate the genetic structure of important mosquito vectors in SSA [190], the use of genetic and morphological techniques for the molecular classification of various and unidentified mosquito vectors in the western Kenyan highlands [150], the characterization of potential *Plasmodium vivax* vectors using molecular and morphological methods in central and eastern Sudan [191], genetic characterization, the composition of species and dispersion maps of mosquito species in Benue State, Nigeria [5], the molecular identification of the *Anopheles nili* group of African malaria vectors, which are recognized to be important malaria carriers in SSA [124], and genetic classification and wing distinctions amongst mosquito vectors in the Akure North Local Government Area, Nigeria, which focuses on the main vector of *Plasmodium* in Africa, the *Anopheles gambiae* complex [188]. Although these suggest that utilizing molecular techniques in vector management is gaining ground in sub-Saharan Africa, this is not without some attending challenges and limitations. Some of the challenges associated with using molecular identification methods for studying mosquito vectors in SSA are as follows: the presence of species complexes in *Anopheles* vectors making it difficult to distinguish between sibling species (isomorphic species) using morphological methods alone [188], the lack of adequate funding for vector control activities in malaria-endemic nations, which limits the implementation of molecular identification methods [14], the need for specialized equipment and expertise to perform molecular identification, which is often unavailable in all regions of SSA [190], the lack of comprehensive databases of molecular markers for all mosquito vector species in sub-Saharan Africa, which can make it difficult to identify unknown or newly emerging vector species [150], and the susceptibility of mosquitoes in sub-Saharan Africa to presently employed vector control techniques needing to be examined rapidly [191] and incorporated into malaria control programs, particularly in the face of the pressing global elimination drive.

## 5. Evidence Supporting the Superiority of DNA-Based Identification of Malaria Vectors over Morphological Identification

Malaria vector species may be identified using molecular and morphological techniques. Most experts believe that molecular identification is more precise and preferable for identifying malaria vector species. This is because molecular identification relies on highly specific identification procedures. On the other hand, morphological identification has a number of drawbacks, such as the possibility of losing a body part during sample collection. Because several malaria vectors have similar phenotypic traits (members of species complex) but differ in their DNA, morphological identification procedures may mistakenly identify species. Utilizing PCR-RFLP analysis of the ITS2, Gao et al. [152] managed to distinguish *Anopheles anthropophagus* and *Anopheles sinensis* species, which are closely related species that were unable to be differentiated morphologically.

Focusing on egg morphology, some species collected in Guangdong were recognized as *Anopheles anthropophagus*; however, when molecular identification was performed, some were classified as *Anopheles*. Also, in a study conducted by Gao et al. [152], some species collected from Liaoning were morphologically identified as *Anopheles anthropophagus* due to having a narrow deck egg, whereas others appeared to be *Anopheles sinensis* because they had wide deck egg. However, PCR-RFLP identified all species collected from Liaoning as *An. Anthropophagus*. Several markers such as *COI*, *5.8S*, *28S*, *16S-rDNA,* and *ITS2* successfully identified mosquitoes of the same complex in different regions across the world [143,144,145,146,147,148,149,150,151]. Despite the limitations of the various sequencing techniques previously outlined, their strengths make them the main instrument employed by geneticists to investigate population genetics. All genetic approaches employed in mosquito identification, including sequencing techniques such as next-generation sequencing, genotyping-by-sequencing, and so on, are claimed to be very specific. As a result, the capacity for molecular characterization of malaria vectors should be expanded, and morphological characterization should not be depended on or approved without molecular approaches. All of this information combined together provides evidence that molecular identification is more accurate than morphological identification; hence, scientists should rely mostly on molecular identification as one of the tools to eradicate malaria.

## 6. Routes to Be Taken to Advance Malaria Vector Control Strategies in Different Endemic Regions and Basic Solutions to Overcome Insecticides’ Resistance and Imported Cases

Since the distribution and prevalence of malaria varies by region, malaria vector control strategies vary by region and the kind of malaria vector responsible for parasite transmission in that region. Malaria has killed an exceptionally high number of human beings worldwide, despite malaria vector control methods. The reason for this is because malaria vectors are resistant to the pesticides utilized. As a result, scientists must investigate the source of resistance and devise new methods for managing malaria vectors. Malaria resistance is not the only issue challenging many regions, such as Europe and the United States; imported cases are also a problem. A possible solution to the cases imported is that cross-border initiatives with government assistance should be strict, not allowing illegal border crossings, and everyone who enters the region legally should be screened for malaria and treated if afflicted. To combat the problem of airplane malaria, the plane and luggage should be treated with pesticides a few hours before the flight.

As mentioned previously, the resistance of malaria vectors to insecticides and other malaria control measures is a major concern in almost all malaria-endemic regions. However, prior studies employed genetic markers to identify some of the genes that cause resistance in different species; hence, such knowledge will serve as fundamental data for medical researchers or chemists to develop treatments or pesticides that will kill vectors with resistant genes. The *Vgsc* gene has been identified as one of the genes that, if mutated, causes resistance [127]. Since earlier research has uncovered how genetically varied wild mosquito species are, how pesticide resistance develops, how malaria is transferred, and how frequently it is subjected to selection advantage, science promises to eradicate malaria in almost all parts of the globe.

## 7. Future Insight and Prediction

The future of malaria vector control lies in the enhanced molecular capacity for accurate species identification. Molecular tools have been developed to identify and confirm mosquito species, enabling early detection of invasive species like *Anopheles Stephensi*. This enhanced molecular capacity will improve mosquito characterization, leading to more accurate control strategies and treatments targeting specific mosquito species, thus contributing to malaria eradication. In addition to molecular tools, the capacity for disease surveillance will significantly benefit from the increased development of more accurate, precise, automated, and high-throughput diagnostic techniques. This will allow for the accurate identification of *Plasmodium spp.* causing malaria in patients, which is crucial for disease epidemiology and surveillance.

While morphological identification is quick and achievable at a reduced cost, molecular identification is preferred for its specificity and sensitivity. To achieve the targeted malaria elimination goal, proper identification of vectors using accurate techniques for effective control measures should be prioritized. Overall, the future of malaria vector control will be shaped by the continued development and implementation of enhanced molecular tools for accurate species identification, which will in turn lead to more effective and targeted control strategies, ultimately contributing to the global effort to eradicate malaria.

## 8. Conclusions

Based on previous studies, the molecular identification of malaria vector species, understanding the life cycle of various *Plasmodium spp.*, and understanding the role of both traditional and molecular control methods can all aid in the elimination of malaria across the globe. Explicit details of mosquito vectors, their habits, vectorial capacity, pesticide resistance, and other transmission-related traits are essential for understanding local transmission and deploying effective treatments. While molecular identification is accurate and desirable, there are still gaps that must be filled. In most places across the world, there are relatively few studies based on the genes that create malaria vector resistance; therefore, further studies concentrating on such genes are still needed. The information gathered in this review demonstrates that the molecular characterization of mosquito species produces more reliable results than morphological characterization; thus, the capacity for employing molecular characterization should be increased. However morphological identification should always be the primary tool for identifying malaria vectors, and its results should always be verified by molecular characterization, which has been shown to be more accurate. Precise identification of malaria vectors would enable the development and assignment of an efficient control strategy, which would ultimately result in the eradication of malaria in endemic regions, provided that morphological–molecular identification of malaria vectors strategy is prioritized.

## Figures and Tables

**Table 1:** Examples of markers normally utilized for identifying *Anopheles* complexes in different countries or regions.

	Markers	Countries/Regions	*Anopheles* Complex Identified	Reference
1.	COI ITS2	Sri Lanka	*An. culicinae* complex	Weeraratne et al. [143]
2.	COI	Australia (Victoria State)	*An. culicidae* complex	Batovska et al. [144]
3.	ITS2 COI	Middle Asia and Kazakhstan	*An. Maculipennis* complex	WHO, [145]
4.	COI ITS2	Portugal	*An. maculipennis* complex, *An. claviger* complex, and *Aedes detritus* complex	Madeira et al. [146]
5.	ITS2 5.8S 28S	India, i.e., Gurugram, Nuh, Alwar, and New Delhi from northern India, Ranchi, Raipur, and Gadhchiroli from central India, Goa, Bangalore, Mangalore, Chennai, and Mysuru from southern India	*An. stephensi*	Mishra et al. [147]
6.	ITS2 16S-rDNA	North-central Nigeria	*An. culicidae*	Iyiola et al. [148]
7.	ITS2 COI	Karama, west Sulawesi, and Indonesia	*An. aconitus*; *An. barbirostris*; *An. karwari*; *An. peditaeniatus*; *An. tessellatus*; *An. vagus; An. kochi; An. flavirostris*; *An. nigerrimus*; and *An. maculatus*	Davidson et al. [149]
8.	ITS2 COI	Kenyan highlands (Nyanza Province)	*An. gambiae and* *An. funestus*	St Laurent et al. [150]
9.	ITS2 D3 28SDomain	Cameroon, Burkina Faso, Ivory Coast. and Senegal	*An. nili*	Kengne et al. [124]
10.	ITS2	South-east Asia (Hanoi suburbs; Hoa Binh; Ninh Binh; Khanh Hoa; Dak Lak; Binh Thuan; Vientiane; Kanchanaburi; Rattanakiry)	*An. minimus*	Van Bortel et al. [151]

## Data Availability

All data regarding this work can be found in the manuscript.
